# Trajectory Identification for Moving Loads by Multicriterial Optimization

**DOI:** 10.3390/s21010304

**Published:** 2021-01-05

**Authors:** Michał Gawlicki, Łukasz Jankowski

**Affiliations:** Institute of Fundamental Technological Research, Polish Academy of Sciences, Pawińskiego 5B, 02-106 Warsaw, Poland; michal.gawlicki1@gmail.com

**Keywords:** structural health monitoring (SHM), moving load, trajectory identification, geometric regularity, multicriterial optimization, load identification, inverse problems, structural mechanics

## Abstract

Moving load is a fundamental loading pattern for many civil engineering structures and machines. This paper proposes and experimentally verifies an approach for indirect identification of 2D trajectories of moving loads. In line with the “structure as a sensor” paradigm, the identification is performed indirectly, based on the measured mechanical response of the structure. However, trivial solutions that directly fit the mechanical response tend to be erratic due to measurement and modeling errors. To achieve physically meaningful results, these solutions need to be numerically regularized with respect to expected geometric characteristics of trajectories. This paper proposes a respective multicriterial optimization framework based on two groups of criteria of a very different nature: mechanical (to fit the measured response of the structure) and geometric (to account for the geometric regularity of typical trajectories). The state-of-the-art multiobjective genetic algorithm NSGA-II is used to find the Pareto front. The proposed approach is verified experimentally using a lab setup consisting of a plate instrumented with strain gauges and a line-follower robot. Three trajectories are tested, and in each case the determined Pareto front is found to properly balance between the mechanical response fit and the geometric regularity of the trajectory.

## 1. Introduction

Aging infrastructure and increasing operational loads require development and implementation of effective methods for structural monitoring [1,2,3]. Within the last two decades, the related field of structural health monitoring (SHM) has witnessed a rapid progress in basic research approaches [4,5], in technology [6], as well as an increasing number of successful practical applications [7,8,9]. This field can be broadly divided into two areas: damage identification and load monitoring. The area of damage identification encompasses approaches that aim at detection, localization, and quantification of structural damages [10] and at health testing [11]. However, this paper belongs to the area of load monitoring, which aims at indirect identification of operational or environmental loads and excitations.

The general goal of load identification is to infer certain characteristics of structural loads in an indirect way, that is, based on recorded structural response and a certain kind of structural model. Such an approach can be opposed to the direct measurement of excitations, which in many applications is not possible or difficult [12]. Load identification problems belong thus to the general class of inverse problems of input identification; they were extensively studied and detailed reviews can be found, for example, in [13,14,15].

In a general, straightforward formulation of the load identification problem, there is usually a conflict between the generality of the unknown load and the limited availability of information. This is a result of two typical, contradictory features of such problems:

- A large number of potential excitation points, that is, the degrees of freedom (DOFs) in which the excitation force can be applied and should be thus identified.
- A limited number of sensors that can be employed to measure the structural response to the unknown excitation and provide information for the identification process.

There is usually insufficient information about the load to enable its full identification, so that the identification problem is not well posed in the sense of Hadamard [16]: the solution is non-unique and numerically ill-conditioned. To ensure the uniqueness, typical approaches restrict the generality of the load to be identified. The unknown force is often assumed to be pointwise and stationary, so that the aim is to identify its time-dependent magnitude only [17]. With the structure remaining in the linear range, such a formulation is basically equivalent to the problem of deconvolution [18], which is known to lead to ill-conditioned numerical formulations [19]. In addition to typical $\ell_{2}$-based regularization methods, the ill-conditioning can also be addressed using $\ell_{1}$-based regularization that assumes a sparse representation in a broad, redundant dictionary [20,21,22,23]. However, the assumption of a stationary pointwise excitation is not directly applicable in the case of unknown moving loads. A typical solution mode in such a case is to assume the trajectory of the moving load to be known [24], and this is an especially natural approach in case of identification of vehicle load from bridge responses [25]. The task can be further reformulated as the problem of identification of vehicle parameters (most importantly mass) instead of the related time-dependent excitation force; this reduces the number of unknown parameters to be identified, but it requires the dynamics of the coupled vehicle–bridge structure to be considered [26,27].

Formulations that aim at simultaneous identification of the locations and time-dependent magnitudes of the unknown forces are rare. A two-step iterative procedure for localization and identification of a single force is proposed in [28]. Force localization is based on a stabilization diagram computed for the regularization parameter, but the assumption of a single stationary force precludes direct application in the case of moving loads. A case with a larger number of unknown forces is considered in [29], which proposes an approach for full time-domain identification of their magnitudes and locations. The sparse Kalman filer is employed, where the spatial locations of the forces are predicted in each step using the relevance vector machine (RVM). In [30], an optimization approach is proposed based on a mixed group regularization scheme: $\ell_{1}$ regularization is used to promote spatial sparsity and determine the locations of the forces, while their spectra are simultaneously identified by means of $\ell_{2}$ regularization. This approach is reformulated in [31] as a second order cone program, which allows an interior point optimization scheme to be used. A related space-frequency mixed multiplicative regularization approach with an informed regularization term is proposed in [32]. The problem of identification of locations and time-domain reconstruction of multiple unknown forces is recently formulated and solved also in the Bayesian framework [33].

It should be emphasized that the unknown forces considered in the above methods, even if multiple, are assumed to be stationary. A time series of such stationary forces of short duration can be used to represent a moving force, and such an approach is investigated in [34], where vehicle positions along a bridge are identified together with the lane number by means of sparsity-based methods. However, the case of a moving load is conceptually different from the case of a set of independent forces. In the case of independently treated forces, no specific spatial distributions or time sequences are promoted a priori. In contrast, in the case of a moving load, the crucial factor is the trajectory of the load, which needs to satisfy certain natural continuity or geometric regularity constraints. These constraints can be exploited in the identification process to provide additional information and ensure more reliable results. Astonishingly, such an approach seems to be unexplored. Consequently, this paper focuses on the case of a moving load and on the problem of trajectory identification using geometric regularity conditions, including an experimental verification in laboratory conditions. The focus on the trajectory contrasts with the usual approaches that concentrate on identification of the time-dependent force magnitude and typically assume the load to be stationary or the trajectory to be known. Earlier attempts included sparsity-based identification of 1D trajectories of moving mass loads on a laboratory beam with transient dynamics [35], but they did not consider the geometric characteristics of the trajectory. Here, a fully two-dimensional case is considered: the unknown load is freely moving in 2D on a plate structure. Typical optimization criteria, based on the mechanical response only, tend to yield erratic and nonphysical trajectories, especially if the instrumentation is limited. It is thus proposed to use concurrently two complementary criteria of very different natures: mechanical and geometric [36]. The former is the typical objective function that quantifies the mechanical response discrepancy, while the latter quantifies certain geometric features of the trajectory such as stability of linear and angular velocities [37]. A multicriterial optimization approach is then used, which effectively allows the erratic response-based trajectory to be numerically regularized with respect to the geometric regularity criteria. The optimization is performed using a specialized state-of-the-art multicriterial genetic algorithm NSGA-II [38,39]. A nonparametric model of the structure is employed in the form of a set of experimentally collected measurements, which allows the tedious processes of finite element model building and updating to be avoided. The approach is experimentally verified in laboratory conditions: a plate instrumented with three strain gauges is used with a line-follower robot in the role of the moving load.

The paper is structured as follows. Section 2 introduces the model of the load and the nonparametric model of the structure. Section 3 describes the proposed multicriterial identification approach, including the response-based objective function and a compound measure of geometric regularity of a candidate trajectory. Numerical implementation is discussed in Section 4, including numerically efficient versions of the objective functions, a binary representation and encoding of the trajectory, and other details required for the implementation of NSGA-II. Finally, Section 5 presents experimental verification in laboratory conditions performed using a plate subjected to a line-follower robot and three example trajectories.

## 2. Moving Load and Nonparametric Structural Model

### 2.1. Moving Load

A force load model is considered. The trajectory of the moving load, that is, the position of the force in time, is denoted by $x\left(t\right)=\left(x_{1}\left(t\right),x_{2}\left(t\right)\right)$, and it is assumed to be confined to a certain two-dimensional domain D on the boundary of the structure,

(1) $$x:\left[0,T\right]\to D\subset R^{2},$$

where *T* is the duration of the considered time interval. The corresponding magnitude of the load is denoted by the non-negative scalar f(t),

(2) f:[0,T]→[0,∞).

### 2.2. Measured and Modeled Response

The response of the structure is measured with *N* sensors, which are assumed to be linear, and their signal, recorded in response to the actual load, is denoted by $\varepsilon^{M}\left(t\right)\in R^{N}$. Unlike the measured response, which is recorded and given a priori to constitute the basis for identification, the modeled structural response depends on the structural model, and it is a function of the moving load as defined in terms of x(t) and f(t). The modeled response is denoted by $\varepsilon \left(x,f\right)\left(t\right)\in R^{N}$.

Under zero initial conditions, the modelled response can be represented in the following form,

(3) ε(x,f)(t)=K(x,f)(t),

where K is a certain operator. Similarly to the sensors, the structure is also assumed to be linear, so that the operator K is linear with respect to the load magnitude *f*. In the general dynamic case, K is a convolution,

(4) $$\varepsilon \left(x,f\right)\left(t\right)=K\left(x,f\right)\left(t\right)=\int_{0}^{t}f\left(\tau \right)k_{x(\tau )}\left(t-\tau \right)\;d\tau ,$$

where $k_{x}\left(\cdot \right)$ is the *N*-dimensional vector of the impulse response functions that describe the responses of the respective sensors to an impulsive excitation applied at the position x∈D. In the the quasi-static case, the operator K simplifies to the following scalar–vector product,

(5) $$\varepsilon \left(x,f\right)\left(t\right)=K\left(x,f\right)\left(t\right)=f\left(t\right)k_{x(t)},$$

where $k_{x}$ is the vector of the sensor responses to a static unit force load applied at the position x. Note that in both cases the set $\left\{k_{x}:x\in D\right\}$ constitutes a nonparametric model of the structure, reduced to the considered sensors and the load domain D. Such a model can be obtained numerically, e.g., using an updated finite element (FE) model, or purely experimentally, as shown in Section 5.2.

## 3. Trajectory Identification

Let the structure at hand be subjected in the time interval [0,T] to a moving load with an unknown trajectory x(t) and an unknown magnitude f(t). Let $\varepsilon^{M}\left(t\right)$ denote the corresponding structural response that was measured by the available sensors. The task of trajectory identification can be formulated as the inverse problem of identification of x(t) based on the available signal $\varepsilon^{M}\left(t\right)$. Such a formulation requires one or more appropriate objective functions to be defined.

In typical formulations of the load identification problem available in literature, the identification is based on a single natural objective function that quantifies the discrepancy between the measured and the modeled response of the structure. In the general case of a freely moving 2D load, as considered here, a direct application of such a formulation tends to yield erratic solutions due to measurement and modeling errors, especially in the limited instrumentation case. As illustrated in Section 5.3, the trivial solutions obtained this way contain obvious, nonphysical spatio-temporal inconsistencies (sudden jumps, widely varying velocity, etc.).

Therefore, in the following subsections, this paper proposes two complementary objective functions to be simultaneously minimized in a multicriterial optimization problem. The functions are of very different natures: mechanical (to quantify the response discrepancy) and geometric (to quantify the geometric irregularity of the trajectory).

### 3.1. Measurement-Based Objective Function

The fundamental objective function has a mechanical character, and it quantifies the discrepancy between the measured response $\varepsilon^{M}\left(t\right)$ of the physical structure and the modeled response ε(x,f)(t) treated as a function of the load trajectory:

(6) $$F_{1}\left(x\right)≔\gamma \;\ln\left(1+\frac{1}{T}\min_{f\geq 0}{∥\Delta \varepsilon \left(x,f\right)∥}_{2}^{2}\right),$$

where γ is a constant normalization factor and the misfit is quantified in terms of the $\ell_{2}$ norm of the response modeling error:

(7) $${∥\Delta \varepsilon \left(x,f\right)∥}_{2}^{2}≔\int_{0}^{T}{∥\varepsilon^{M}\left(t\right)-\varepsilon \left(x,f\right)\left(t\right)∥}^{2}dt.$$

The internal minimization in Equation (6) with respect to the non-negative load magnitude *f* expresses the fact that this paper focuses on the identification of the trajectory only. Due to the assumed linearity of the structure, see Equation (4) or (5), such a minimization constitutes a quadratic programming problem, which is relatively easy to be solved, especially in the discretized time setting.

The normalization coefficient γ in Equation (6) ensures that the minimum value of $F_{1}$ is one:

(8) $$\gamma ≔\frac{1}{\min_{x}\;\ln\left(1+\frac{1}{T}\min_{f\geq 0}{∥\Delta \varepsilon \left(x,f\right)∥}_{2}^{2}\right)}.$$

It should be noted that a formal mathematical formulation of minimization in Equations (6) and (8) would require specification of the function spaces that *f* and x belong to. However, the trajectory is, in practice, represented in the discrete form of a finite sequence of *n* points to be interpolated, which simplifies the actual search spaces to ${\left[0,\infty \right]}^{n}$ and $D^{n}$, respectively, and allows the related mathematical intricacies to be neglected.

### 3.2. Geometric Regularity of the Trajectory

The second objective function is used to quantitatively express the expectation that reasonable trajectories should be characterized by some degree of geometric regularity. It takes the following compound form,

(9) $$F_{2}\left(x\right)≔\alpha F_{21}\left(x\right)+\beta F_{22}\left(x\right),$$

where $F_{21}$ and $F_{22}$ quantify two different geometric characteristics of the trajectory: angular wiggling and relative stability of linear velocity, respectively.

*Angular wiggling $F_{21}$.* The component $F_{21}$ is intended to limit excessive changes of the angular velocity of the load:

(10) $$F_{21}\left(x\right)≔\frac{1}{T}\int_{0}^{T}\ln\left(1+{\dot{\theta }}^{2}\left(t\right)\right)dt,$$

where $\dot{\theta }\left(t\right)$ is the angular velocity of the moving load,

(11) $$\dot{\theta }\left(t\right)≔\frac{\left|\det\left[\dot{x}\left(t\right)\;\ddot{x}\left(t\right)\right]\right|}{\sqrt{10^{-3}+{∥\dot{x}\left(t\right)∥}^{4}}}.$$

The term $10^{-3}$ present in the denominator of Equation (11) is a small term added to avoid numerical indeterminacy for temporarily stationary loads, which otherwise would occur whenever $\dot{x}\left(t\right)=0$.

*Stability of linear velocity $F_{22}$.* The component $F_{22}$ is intended to limit excessive variability of the linear velocity of the load. It is defined in analogy to the coefficient of variation as the root mean square (rms) linear velocity normalized with respect to the mean value,

(12) $$F_{22}\left(x\right)≔\frac{\mathrm{rms}∥\dot{x}∥}{\mathrm{mean}∥\dot{x}∥},$$

where

(13) $$\begin{array}{cc} \mathrm{rms}∥\dot{x}∥ & =\sqrt{\frac{1}{T}\int_{0}^{T}{∥\dot{x}∥}^{2}\left(t\right)\;dt}, \end{array}$$

(14) $$\begin{array}{cc} \mathrm{mean}∥\dot{x}∥ & =\frac{1}{T}\int_{0}^{T}∥\dot{x}\left(t\right)∥\;dt. \end{array}$$

The weighing coefficients α and β in Equation (9) encode the relative importance of $F_{21}$ and $F_{22}$, respectively. Their numeric values are selected here to equalize the relative importance of $F_{21}$ and $F_{22}$ in the case of the trivial $F_{1}$-optimum trajectory $x_{F_{1}}\left(t\right)$:

(15) $$\begin{array}{cc} \alpha ≔ & \frac{1}{2}\frac{1}{F_{21}\left(x_{F_{1}}\right)}, \end{array}$$

(16) $$\begin{array}{cc} \beta ≔ & \frac{1}{2}\frac{1}{F_{22}\left(x_{F_{1}}\right)}, \end{array}$$

where

(17) $$x_{F_{1}}≔\arg\min_{x}F_{1}\left(x\right).$$

As discussed at the end of Section 3.1, the trajectory x is in practice represented in the discrete form of *n* points, and thus the minimization in Equation (17) is actually performed in $D^{n}$.

### 3.3. Multicriterial Optimization and Pareto Front

The trajectory identification problem is formulated as a multicriterial optimization problem. The aim is to find the trajectories x that simultaneously minimize both objective functions $F_{1}$ and $F_{2}$:

(18) $$\begin{array}{cc} \mathrm{minimize} & \;F_{1}\left(x\right),F_{2}\left(x\right)\\ w.r.t & \;x\\ \mathrm{subject}\;\mathrm{to} & \;x(t)\in D\;\;\mathrm{for}\;\;t\in [0,T]. \end{array}$$

As in case of Equations (8) and (17), the actual search space in Equation (18) is in practice simplified to $D^{n}$. The solution of such a multicriterial optimization problem is usually non-unique [40]. It consists of nondominated trajectories, which correspond to the Pareto front in the $\left(F_{1},F_{2}\right)$ space. These trajectories are optimum in the sense that none of them can be improved with respect to both objective functions. By investigating the nondominated solutions along the Pareto front, one can trace the influence that the two objective functions have on the optimum trajectory, and ultimately find the balance between them.

## 4. Numerical Optimization

The multicriterial minimization problem in Equation (18) is a variational problem that seems to be intractable by classical analytical means of variational calculus. Moreover, measurement data have in practice always a discrete character. Therefore, the general continuous formulation presented in Section 2 and Section 3 should be ultimately discretized and solved numerically. This section describes such a discretization and other prerequisites for numerical optimization.

### 4.1. Optimization Algorithm

**Figure 1:**
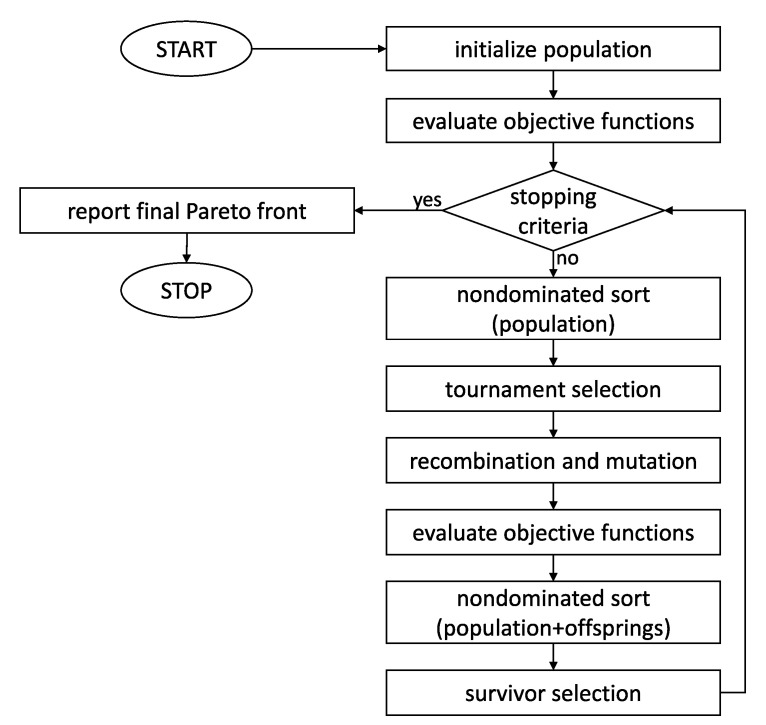
Flowchart of computations: NSGA-II algorithm [38] applied to the trajectory identification problem.

The considered objective functions have relatively complex, multimodal characters. In terms of the optimization algorithm, a natural choice is thus a global search approach. There is a relatively small family of specialized multiobjective global search algorithms [39], and the nondominated searching genetic algorithm (NSGA-II) is used here due to its in-built preference for uniformly populated Pareto fronts [38]. As is typical for genetic algorithms, each trajectory is represented in the binary form, and typical forms of mutation and crossover operators are considered. The specific flowchart of computations is shown in Figure 1. The block “nondominated sort” convert the pairs of objective functions into a single ranking based on Pareto front ranks and crowding distance, to be used for parent and survivor selection [38]. As a stopping criterion, the maximum number of 10,000 generations is used.

### 4.2. Trajectory Representation and Encoding

NSGA-II, as other genetic algorithms, operates on populations of individuals. Each individual represents a trajectory, which in the general continuous formulation is a function, see Equation (1). However, for optimization purposes and because of the discrete character of the measurement data, it is represented in practice by a finite sequence of *n* points sampled from the loading domain D at uniform time intervals,

(19) $$\begin{array}{cccc} x & ∼\left\{x_{1},x_{2},\ldots ,x_{n}\right\}\in D^{n}, & \mathrm{where}\;x_{i} & =x\left(\frac{i-1}{n-1}T\right). \end{array}$$

If necessary, the continuous form of the trajectory can be approximated by performing a spline-based interpolation of the sequence $\left\{x_{1},x_{2},\ldots ,x_{n}\right\}$. The trajectory is assumed to be 2D, that is $D\subset R^{2}$, and in the discretized form it is consequently represented by a sequence of 2n real numbers,

(20) $$x∼\left\{u_{1},u_{2},u_{3},u_{4},\ldots ,u_{2n-1},u_{2n}\right\}\in R^{2n},$$

where $x_{i}=\left(u_{2i-1},u_{2i}\right)$. In applications considered in this paper, the loading domain D is rectangular, so that $u_{i}$ belongs to a certain interval, $u_{i}\in \left[u_{i0},u_{i1}\right]$. For encoding purposes, each of these intervals is uniformly discretized into $2^{n_{\mathrm{bit}}}$ points, which are then encoded using the Gray encoding scheme (reflected binary code [41]) in $n_{\mathrm{bit}}$ bits $\left(g_{i1},g_{i2},\ldots ,g_{in_{\mathrm{bit}}}\right)\in {\left\{0,1\right\}}^{n_{\mathrm{bit}}}$:

(21) $$u_{i}≔u_{i0}+\frac{u_{i1}-u_{i0}}{2^{n_{\mathrm{bit}}}-1}\sum_{j=1}^{n_{\mathrm{bit}}}2^{j-1}b_{ij},$$

where $\left(b_{i1},b_{i2},\ldots ,b_{in_{\mathrm{bit}}}\right)$ is the standard binary encoding that can be obtained from the Gray encoding in the following way,

(22) $$\begin{array}{cc} \; & b_{i1}≔g_{i1}\\ \; & \mathrm{for}\;\;j≔2\;\;\mathrm{to}\;\;n_{\mathrm{bit}}\;\;\mathrm{do}\\ \; & \;\mathrm{if}\;\;g_{ij}=1\;\;\mathrm{then}\;\;b_{ij}≔\mathrm{not}\left(b_{i(j-1)}\right)\\ \; & \;\mathrm{else}\;\;b_{ij}≔b_{i(j-1)} \end{array}$$

Finally, all these $n_{\mathrm{bit}}$-bit representations are stacked together to form a $\left(2nn_{\mathrm{bit}}\right)$-bit binary encoding of the entire discrete trajectory.

### 4.3. Genetic Operators and Initial Population

The usual 2-way deterministic tournament selection is applied to select the individuals for mating, crossover and mutation. As each individual (a trajectory) is represented and encoded in the standard binary way, typical recombination and mutation operators can be used. The probability of mutation for each bit is ${\left(nn_{\mathrm{bit}}\right)}^{-1}$ and the crossover probability for each mating pair is 0.5.

The NSGA-II, as any other genetic algorithm, requires an initial population to be defined. Here, it is the one-element set that consists of the trivial $F_{1}$-optimum trajectory $x_{F_{1}}$. Such a a choice accelerates the search, as it allows the population to evolve from the trajectories that offer a reasonable fit to the physical measurements. All subsequent generations consist of 100 individuals.

### 4.4. Objective Functions

The objective functions defined in Equations (6) and (9) are formulated in terms of continuous and smooth trajectories. However, only discrete trajectories are available in practice due to the discrete character of the measurement data. The continuous formulations of the objective functions thus need to be accordingly discretized to be computable based directly on the discrete representation of the trajectory, that is, the sequence $\left\{x_{1},x_{2},\ldots ,x_{n}\right\}$, see Equation (19). In particular, the measurement-based objective function $F_{1}$ is estimated by replacing in Equation (7) the integral with the corresponding sum of samples:

(23) $$F_{1}\left(x\right)\approx \ln\left(1+\frac{1}{n}\min_{f\geq 0}\sum_{i=1}^{n}{∥\varepsilon_{i}^{M}-\varepsilon_{i}\left(x,f\right)∥}^{2}\right),$$

where the measured and modeled responses are sampled as in Equation (19),

(24) $$\begin{array}{cc} \varepsilon_{i}^{M}≔ & \varepsilon^{M}\left(\frac{i-1}{n-1}T\right), \end{array}$$

(25) $$\begin{array}{cc} \varepsilon_{i}\left(x,f\right)≔ & \varepsilon \left(x,f\right)\left(\frac{i-1}{n-1}T\right). \end{array}$$

The objective function $F_{2}$ that expresses the geometric regularity of the trajectory is a linear combination of two components, $F_{21}$ and $F_{22}$, see Equations (9), (10), and (12). The former component quantifies the angular wiggling, and it is discretized as follows,

(26) $$F_{21}\left(x\right)\approx \frac{1}{n-2}\sum_{i=2}^{n-1}\ln\left(1+{\left(\Delta \theta_{i}\frac{n-2}{T}\right)}^{2}\right),$$

where $\Delta \theta_{i}$ is the angle between the vectors $x_{i-1}x_{i}$ and $x_{i}x_{i+1}$,

(27) $$\Delta \theta_{i}≔\arccos\frac{{\left(x_{i}-x_{i-1}\right)}^{T}\left(x_{i+1}-x_{i}\right)}{∥x_{i}-x_{i-1}∥∥x_{i+1}-x_{i}∥}.$$

The latter component, $F_{22}$, quantifies the variability of the linear velocity. In the discrete setting, the involved integrals are straightforwardly represented as follows,

(28) $$F_{22}\left(x\right)\approx \frac{\sqrt{\frac{1}{n-1}\sum_{i=1}^{n-1}{∥x_{i+1}-x_{i}∥}^{2}}}{\frac{1}{n-1}\sum_{i=1}^{n-1}∥x_{i+1}-x_{i}∥}.$$

## 5. Experimental Verification

### 5.1. Experimental Test Stand

**Figure 2:**
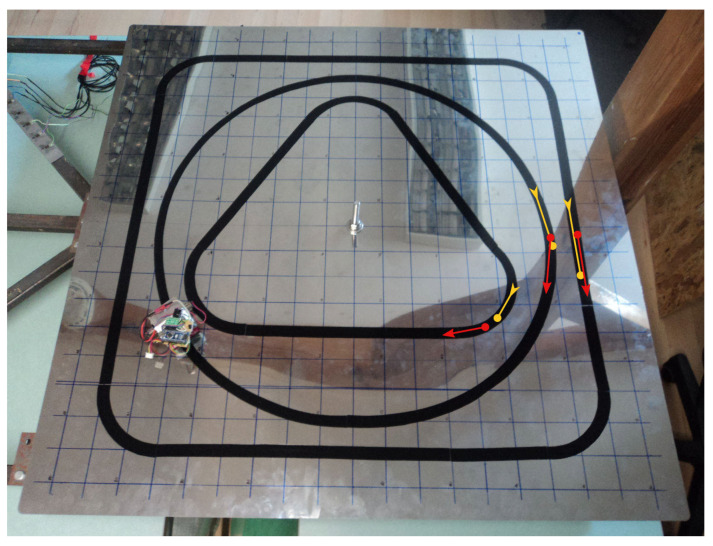
The laboratory test stand: a 1 m × 1 m × 0.5 mm steel plate with a line-follower robot and three test trajectories. The beginning and the end points of each trajectory are marked with red and yellow arrows, respectively. The square and circle trajectories were covered twice, while the triangle trajectory was covered three times.

**Figure 3:**
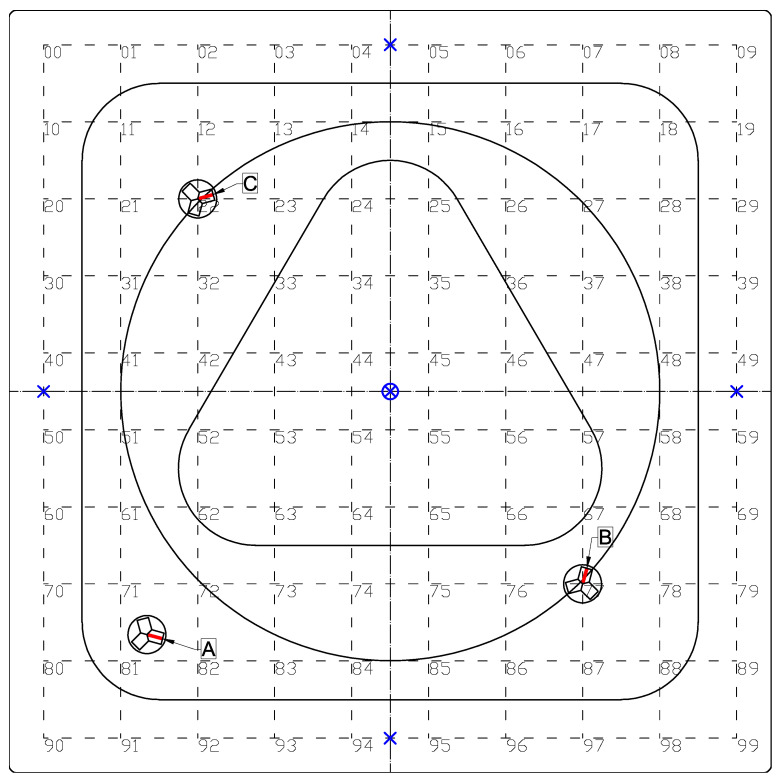
Scheme of the plate: placement of the strain sensors (A, B, and C) on the bottom face, supports (× pin support, ⊗ fixed support), and spatial discretization into a 10 × 10 point grid.

The proposed approach was verified using a laboratory testing stand shown in Figure 2. It consisted of a steel plate, 1 m × 1 m × 0.5 mm in dimensions, pointwise supported in the middle and near the edges, and a line-follower robot with a track width of 28 mm. The moving load was applied in the form of the weight of the robot. Its mass was 0.302 kg, and it used a set of optical sensors to follow one of the three test trajectories shown in the photo (a square, a circle and a triangle) with a constant velocity of about 9 cm/s. The plate was instrumented with three strain gauges, which were denoted A, B, and C, and placed as shown in Figure 3. They were fixed at the bottom face of the plate to avoid collision with the robot and damage.

### 5.2. Responses to Test Trajectories and Nonparametric Model of the Plate

**Figure 4:**
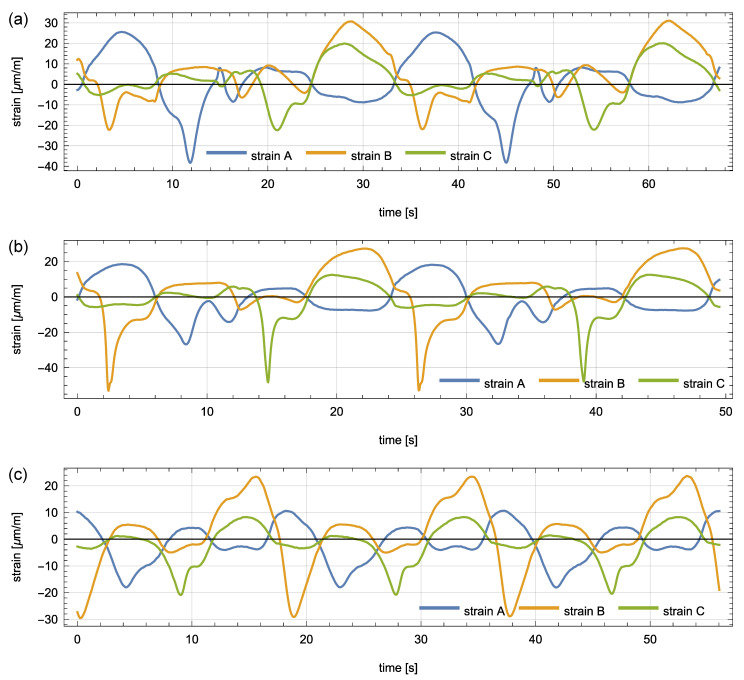
Recorded responses of the three involved strain sensors to the moving load (line-follower robot) following the test trajectories: (**a**) the square trajectory, (**b**) the circular trajectory, and (**c**) the triangle trajectory.

The robot followed the three test trajectories shown in Figure 2 and Figure 3: twice for the square and the circle trajectories, and three times for the shortest triangle trajectory. The beginning and end points of each trajectory are marked in Figure 2 with a red and a yellow arrow, respectively. The corresponding measurement signals of the three strain sensors were recorded and plotted in Figure 4. A clear quasi-static character of the response can be observed. It suggests that the load is also quasi-static, so that Equation (5) can be used to model the response of the plate. Consequently, the nonparametric model of the plate has the form of a set of vectors $\left\{k_{x}\in R^{3}:x\in D\right\}$ that collects the responses of the three sensors to a unit load applied within the load domain D (the plate). This set was constructed experimentally as follows.

**Figure 5:**
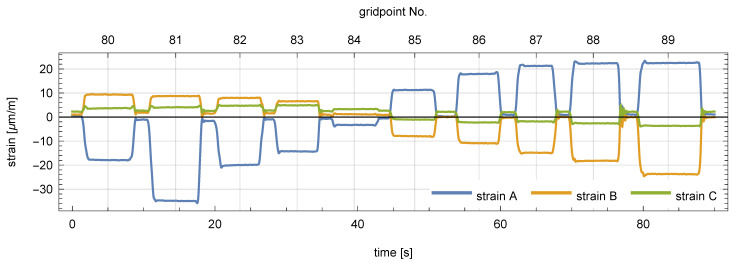
Recorded responses of the three involved strain sensors to the weight of a 0.265 kg mass placed in successive points of the grid (points No. 80 to 89).

**Figure 6:**
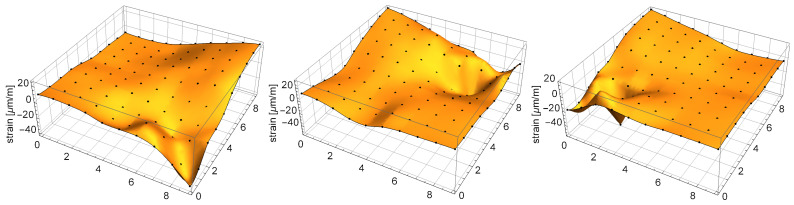
Response surfaces that describe static sensor responses to the constant gravity load of a 0.265 kg mass placed on the plate: (**a**) sensor A; (**b**) sensor B; (**c**) sensor C. The surfaces are interpolated based on the explicitly marked 10 × 10 point grid.

1. The plate was discretized into a 10 × 10 point grid with 10 cm × 10 cm cells, as shown in Figure 3.
2. The constant gravity load of a 0.265 kg mass was applied successively in all 100 points of the grid, and the responses of the sensors were recorded. A fragment of the measurement signal (load in points No. 80 to 89) is shown in Figure 5. A limited degree of nonlinearity can be observed in the responses of the sensors: a small drift of the readings in the unloaded state (bias drift) and a small relaxation-like behavior, which can be probably linked to the sensor–plate adhesive layer. Such effects increase the measurement error, and although they are undesirable in applications, they helped here to test the robustness of the proposed method.
3. Finally, the response vectors corresponding to the 100 grid points were extracted and spline-interpolated in 2D to form the continuous response surfaces and the nonparametric model $\left\{k_{x}\in R^{3}:x\in D\right\}$. The three interpolated response surfaces are shown in Figure 6.

### 5.3. The Trivial $F_{1}$-Optimum Trajectories

The recorded responses (Figure 4) were resampled at a frequency of 1 Hz. To obtain the initial solution for multicriterial optimization, the trivial trajectories were computed by minimization of the measurement-based objective $F_{1}$ in Equation (23). This is equivalent to the minimization of the quadratic discrepancy measure with respect to the discrete representation $\left\{x_{1},x_{2},\ldots ,x_{n}\right\}$ of the trajectory x. The structure is linear, so that given the trajectory x, the internal minimization in Equation (23) with respect to the load magnitude *f* is a relatively simple quadratic programming problem. Given Equation (5), it further decouples into a series of separate independent one-variable quadratic minimization problems, one for each successive load position $x_{i}$. Therefore, the minimization of $F_{1}$ with respect to the trajectory can be also decoupled into a series of simple independent optimization problems with respect to $x_{i}$. In the discrete setting, they can be solved even by a brute search over all possible $2^{2n_{\mathrm{bit}}}$ load positions: in this manuscript $n_{\mathrm{bit}}=6$, which yields a limited number of 4096 one-variable straightforward quadratic minimization problems.

**Figure 7:**
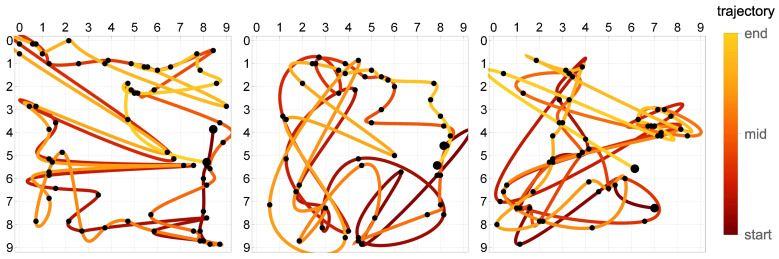
The trivial $F_{1}$-optimum trajectories. The black dots correspond to the 1 Hz time sampling, while the line in-between is the 3rd-order spline interpolation. The actual trajectories were: (**a**) square, (**b**) circle, and (**c**) triangle.

The $F_{1}$-optimum trajectories were computed and plotted in Figure 7. The black dots mark the discrete trajectory points $\left\{x_{1},x_{2},\ldots ,x_{n}\right\}$ and correspond to the 1 Hz sampling rate, while the smooth lines in-between the dots are their 3rd-order spline interpolations. It is clear that these trajectories are indeed trivial in the sense that they are too erratic to be reasonable, physical and useful. This confirms the necessity of the proposed multicriterial optimization approach. The geometric criterion $F_{2}$ can be used to balance $F_{1}$ and alleviate the detrimental influence of all involved errors (measurement errors, sensor nonlinearities, modeling errors, etc.) and the limited availability of information.

### 5.4. Multicriterial Identification of Test Trajectories

**Figure 8:**
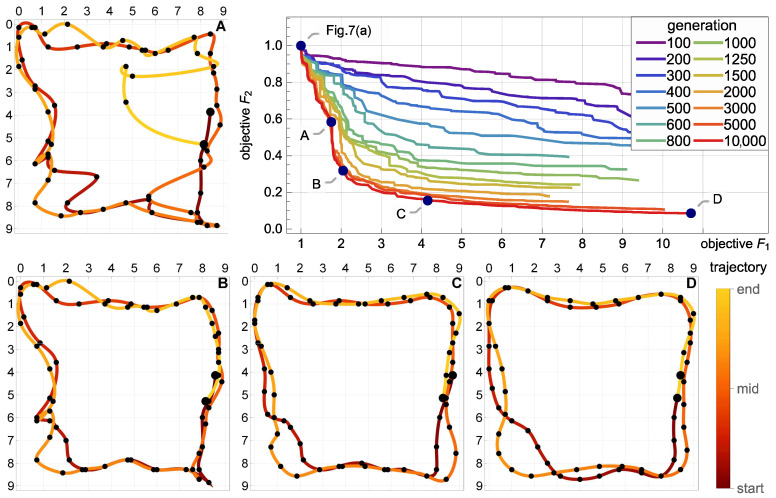
Multicriterial identification of the square trajectory: advancement of the Pareto front and four specific trajectories along the final front (A, B, C and D). The top left dot on the Pareto front corresponds to the trivial $F_{1}$-optimum trajectory shown in Figure 7a. The actual trajectory is shown in Figure 2.

**Figure 9:**
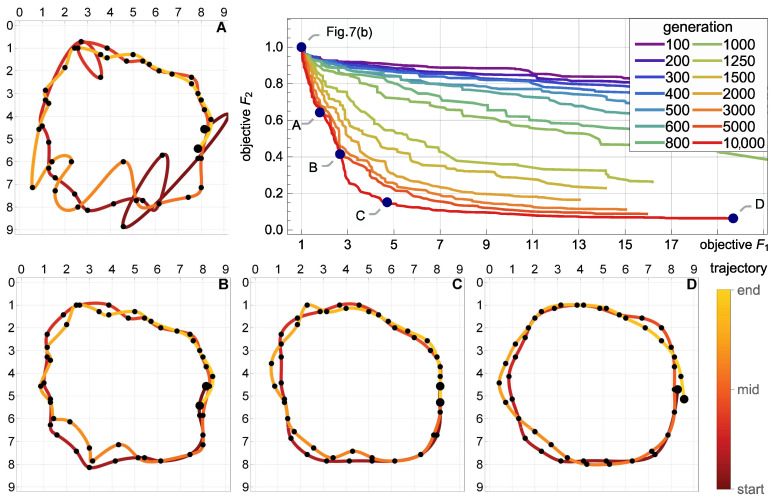
Multicriterial identification of the circle trajectory: advancement of the Pareto front and four specific trajectories along the final front (A, B, C and D). The top left dot on the Pareto front corresponds to the trivial $F_{1}$-optimum trajectory shown in Figure 7b. The actual trajectory is shown in Figure 2.

**Figure 10:**
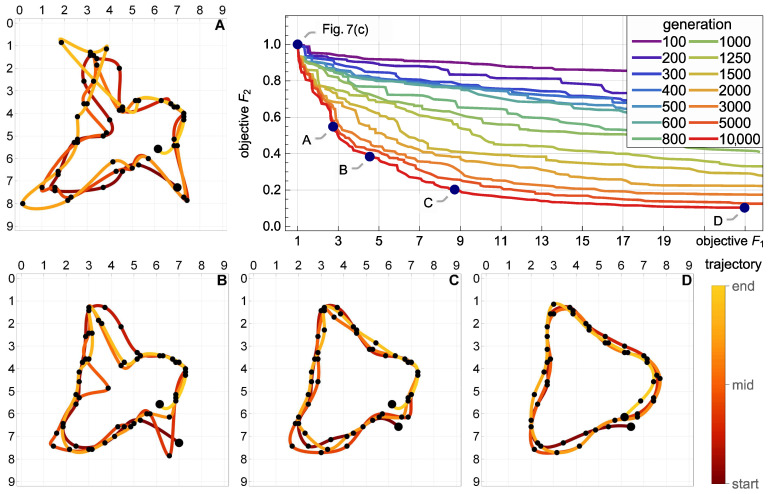
Multicriterial identification of the triangle trajectory: advancement of the Pareto front and four specific trajectories along the final front (A, B, C and D). The top left dot on the Pareto front corresponds to the trivial $F_{1}$-optimum trajectory shown in Figure 7c. The actual trajectory is shown in Figure 2.

Given the erratic nature of the trivial trajectories, the proposed multicriterial optimization scheme was applied to balance $F_{1}$ against the geometric objective $F_{2}$ and to determine the Pareto front. Up to 10,000 generations were evolved. Figure 8, Figure 9 and Figure 10 present the results obtained for the square, circle, and triangle trajectory, respectively. In its top right part, each figure shows the advancement of the Pareto front in the course of the optimization process. Dark blue dots mark five specific trajectories along the final Pareto front. The top left dot corresponds to the respective trivial trajectory, based on $F_{1}$ only and shown in Figure 7. The successive trajectories along the Pareto front (denoted by A, B, C, and D) illustrate the increasing influence of the objective $F_{2}$ and the effect of the geometric regularization it imposes on the solution. These trajectories are shown to the left and below the Pareto plots in Figure 8, Figure 9 and Figure 10. The actual trajectories of the line-follower robot are shown in Figure 2: in each case, their important characteristics were reasonably faithfully identified. It can be seen that at least a qualitative identification of the trajectory was clearly possible.

**Figure 11:**
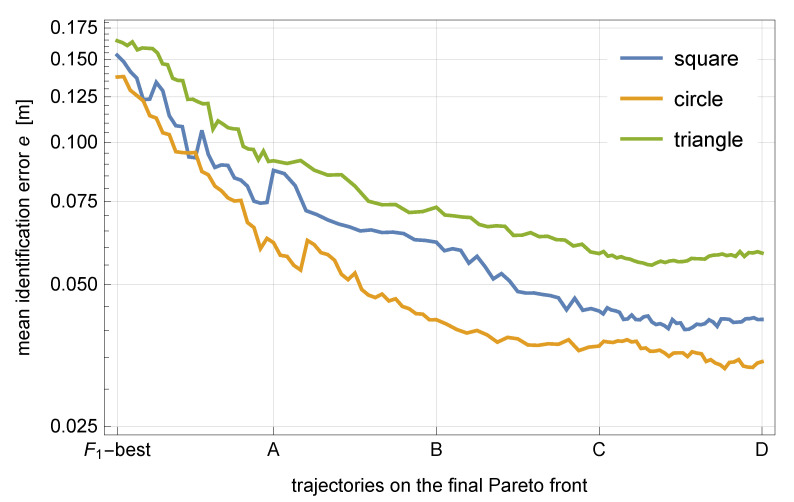
Mean identification error for the trajectories on the final Pareto fronts. Labels “A” to “D” correspond to the labels in Figure 8, Figure 9 and Figure 10, while “$F_{1}$-best” denotes the trivial $F_{1}$-best trajectory.

The accuracy of identification can be assessed quantitatively by computing the mean absolute error of the identified trajectory, which is defined as follows,

(29) $$e\left(x,x^{\mathrm{true}}\right)≔\frac{1}{n}\sum_{i=1}^{n}∥x_{i}-x_{i}^{\mathrm{true}}∥,$$

where x and $x^{\mathrm{true}}$ denote, respectively, the identified and the true trajectory, and *i* indexes the time steps. The three considered true trajectories were compared with the identified trajectories from the respective final Pareto fronts. The results are plotted in Figure 11.

Incorporation of the geometric regularity in the optimization process allows the identification error to be significantly decreased to the range of 30 mm to 60 mm, which is already comparable with the track width of the robot (28 mm) and better than the spatial discretization of the plate used to build its nonparametric response surface model (100 mm). It should be emphasized that in all three cases the error decreases along the Pareto front, although the response residuum increases, as quantified by $F_{1}$. This confirms the insufficiency of the measurement-based criterion and confirms the beneficial regularizing effect of the proposed criterion based on geometric regularity. The obtained level of accuracy, as well as the fact that the identified trajectories cover the entire spectrum of geometric regularity (from erratic to very regular), also suggest that the discretization time step is properly selected to the actual experimental setup.

### 5.5. Compound Trajectories

**Figure 12:**
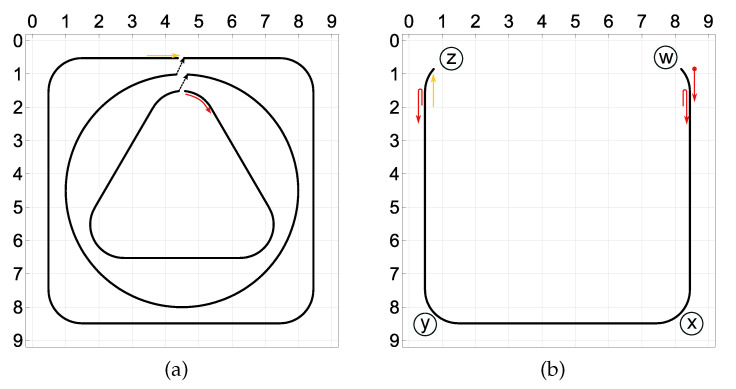
Two compound trajectories constructed using the three basic experimental trajectories: (**a**) The load follows each of the basic trajectories once. (**b**) The load follows three times the U-shaped path along the points w–x–y–z–y–x–w–x–y–z.

In order to test the proposed approach using more complex trajectories, the experimentally measured responses to the three basic trajectories (square, circle, and triangle) were used to construct the two following compound trajectories along with the corresponding responses of the strain sensors:

1. Figure 12a: the moving load starts at the top of the plate and follows clockwise the triangle, circle, and the square trajectory, each of them once. This trajectory has two small discontinuities at the top of the plate which occur when the load switches the basic trajectory.
2. Figure 12b: the moving load starts at the upper right corner of the plate and follows a U-shaped trajectory, which is composed of three segments of the basic square trajectory. The path is followed three times, along the points w–x–y–z–y–x–w–x–y–z, with two sharp U-turns at the points w and z.

**Figure 13:**
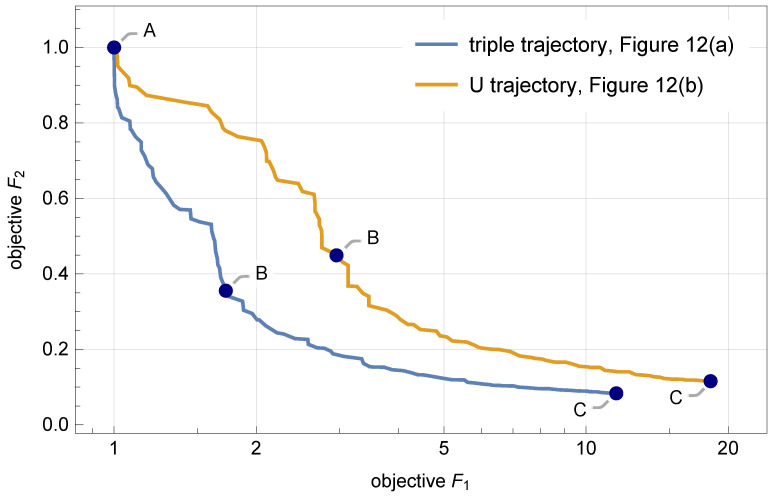
The final Pareto fronts obtained from NSGA-II for the two compound trajectories shown in Figure 12. Three specific points on each front are labeled “A”, “B”, and “C”, and the corresponding identified trajectories are shown in Figure 14 and Figure 15.

**Figure 14:**
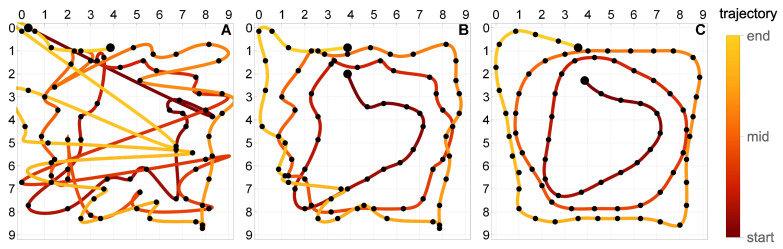
Identification of the compound triple trajectory shown in Figure 12a. The trajectories A, B and C correspond to the three points marked on the respective Pareto front in Figure 13.

**Figure 15:**
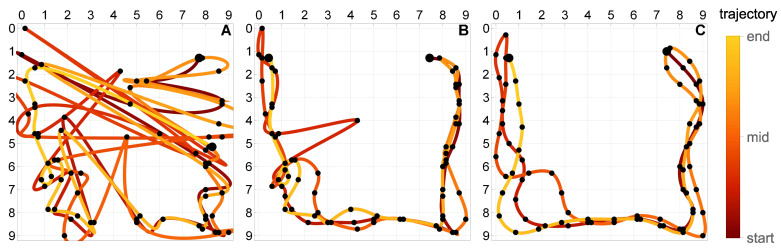
Identification of the compound U trajectory shown in Figure 12b. The trajectories A, B and C correspond to the three points marked on the respective Pareto front in Figure 13.

**Figure 16:**
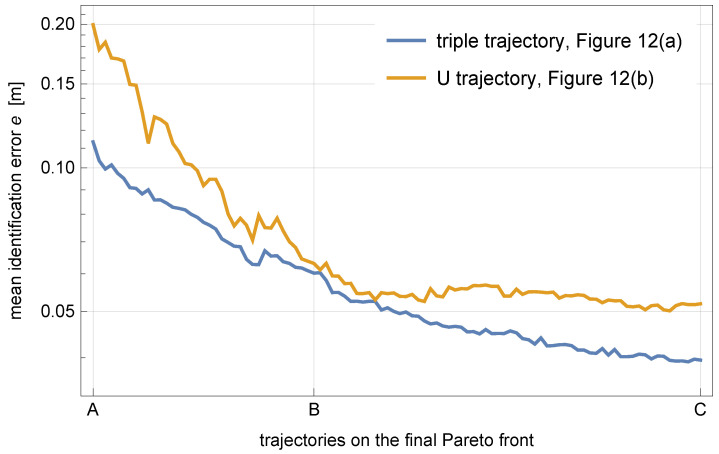
Mean identification error of the trajectories on the final Pareto front obtained for the compound cases shown in Figure 12. Labels “A” to “C” correspond to the labels in Figure 13, Figure 14 and Figure 15.

The proposed multicriterial identification approach was applied to identify these two trajectories based on the constructed sensor responses. The final Pareto fronts obtained from the NSGA-II are plotted in Figure 13. Three specific points on each Pareto front are labeled “A”, “B”, and “C”, and the corresponding identified trajectories are shown in Figure 14 and Figure 15. The mean identification error, defined in Equation (14), was calculated for all identified trajectories, along both Pareto fronts, and plotted in Figure 16. Similarly as in the case of the three basic trajectories, the proposed approach properly identified qualitative characteristics of both compound trajectories. In quantitative terms, the geometric regularity criterion decreased the mean identification error to the level of 40 mm to 60 mm.

## 6. Conclusions

This paper has proposed a multicriterial approach for identification of moving load 2D trajectory. Two complementary criteria of very different natures are used: (1) a mechanical criterion aimed at fitting the modeled and measured structural responses and (2) a newly proposed geometric criterion aimed at promoting physically reasonable trajectories. As a result, otherwise erratic trajectories are numerically regularized with respect to geometrical consistency measures that express typical or expected geometric features of trajectories. The approach was verified experimentally using a laboratory test stand with a 1 m × 1 m plate structure and a moving line-follower robot. All tested trajectories were successfully identified in qualitative and quantitative terms. Introduction of the geometric criterion allowed the mean identification error to be decreased 3–4 times to the level of 30 mm to 60 mm, which was already comparable with the track width of the robot (28 mm). For optimization, a specialized state-of-the-art multiobjective genetic algorithm NSGA-II was used.

The discussed problem and the proposed approach generate a number of challenging research tasks. This includes natural direct continuations, such as identification of the load magnitude besides its trajectory and multiple trajectory identification in case of several concurrently moving loads, as well as applications to human-induced loads [42,43] and other types of excitations [44]. There are also several further related open problems: optimum placement of available sensors [45,46], determination of the optimum load trajectory and/or magnitude for the purpose of damage identification [47,48], the coupled problem of online trajectory identification and optimum semi-active structural control [49], and effective utilization of substructural identification approaches [50].

## Data Availability

The data is available on request from the corresponding author, after approval by the Institute of Fundamental Technological Research, Polish Academy of Sciences.
